# Individual Differences in Risk and Protective Factors: The Role of Self-Compassion Components among Emergency Responders

**DOI:** 10.3390/bs14030178

**Published:** 2024-02-25

**Authors:** Ilaria Colpizzi, Celeste Berti, Claudio Sica, Virginia Alfei, Corrado Caudek

**Affiliations:** 1 Health Sciences Department, Università degli Studi di Firenze, 50139 Firenze, Italy; claudio.sica@unifi.it; 2 NEUROFARBA Department, Università degli Studi di Firenze, 50139 Firenze, Italy; celeste.berti@gmail.com (C.B.); virginia.alfei2@unibo.it (V.A.)

**Keywords:** self-compassion, individual differences, rescue workers, protective factors, risk factors, coping strategies, resilience

## Abstract

This study investigates individual differences in protective and risk factors among rescue workers (RWs), particularly Red Cross members, to optimize well-being and job performance under high-stress conditions. Employing a person-centered approach, two psychological profiles were identified: an adaptively resilient profile and a maladaptively vulnerable profile, characterized by distinct personality traits, coping methods, life events, and social support networks. A notable external criterion, self-compassion, discerned the profiles with maladaptively vulnerable individuals who exhibited higher self-judgment, social isolation, and emotional over-identification. The study also examined the impact of job roles on these profiles, discovering a prevalence of adaptive resilience among drivers, contrasting with team members who displayed maladaptive resilience and lower self-compassion scores. These insights suggest a nuanced method for identifying RWs who require specialized support, proposing tailored interventions, especially those enhancing self-compassion. The study, through an extensive psychological metric analysis, provides a deeper comprehension of resilience and vulnerability among RWs. This research highlights the importance of recognizing individual differences in protective and risk factors, thereby contributing to the enhancement of mental health and resilience in high-stress professions.

## 1. Introduction

Rescue workers (RWs) and healthcare professionals are repeatedly exposed to potentially traumatic events and to the emotional distress of others, a reality that puts them at heightened risk for conditions such as burnout [1,2], compassion fatigue [3], and even Post-Traumatic Stress Disorder (PTSD) [4,5]. Given these occupational vulnerabilities, understanding and promoting factors that fortify their resilience becomes a matter of critical importance [6,7].

Resilience, understood as the capacity for positive adaptation in the midst of significant challenges, is influenced by a nuanced interplay between protective and risk factors. Protective factors serve to mitigate the adverse effects of stressful situations, whereas risk factors increase the probability of negative, maladaptive outcomes. In professions like rescue work, resilience is not merely a desirable trait but a critical asset, as it enhances an individual’s capacity for self-preservation and effective coping during disruptive, traumatic, or potentially life-altering events [8].

Self-compassion remains a conspicuously understudied dimension, with significant potential to influence resilience. The concept of the “self” has emerged as an increasingly central factor in individual differences related to stress management [9]. Self-compassion, defined by a nurturing relationship with oneself, not only elevates mental well-being but also confers a protective shield against psychological disorders [10,11,12].

This study is designed to examine the relationship between individual differences in protective and risk factors among RWs and their propensity to employ self-compassion as a coping strategy. Specifically, the study seeks to achieve the following:

1. Identify distinct individual difference “profiles” based on combinations of protective and risk factors and examine their respective associations with varying levels of self-compassion; and 
2. Determine which facets of self-compassion are predominantly linked to maladaptive resilience profiles in RWs.

### 1.1. Individual Differences in Protective and Vulnerability Factors

The growing literature on the resilience of emergency responders underscores the complex interplay between various protective and vulnerability factors that influence their post-traumatic responses [13,14]. Such factors span multiple dimensions, each exerting a unique influence on psychological resilience and overall well-being [15]. Factors like robust physical health, specific demographic features, and positive personality traits, such as optimism, serve to augment resilience. Conversely, factors like youth, being female, or a prior history of trauma can attenuate it. Moreover, the employment of adaptive coping strategies and strong social support networks enhances resilience, while maladaptive coping mechanisms and emotional volatility undermine it. Specialized training programs have also been shown to bolster resilience among emergency responders [16].

Despite the critical nature of these factors, self-compassion remains a conspicuously understudied dimension, with significant potential to influence resilience. Self-compassion can be understood as the intrapersonal counterpart to compassion directed towards others. It embodies a warm, caring, empathetic, and nonjudgmental attitude toward oneself, particularly during challenging or distressing periods. This approach is also motivated by a desire to mitigate one’s own suffering. According to Neff (2003) [17], self-compassion is conceptualized through three main facets: (1) Self-Kindness, which involves a friendly and understanding demeanor towards oneself when confronted with stress or failure, contrasting with self-criticism; (2) Common Humanity, which captures the recognition that individual suffering is not isolated but part of a universal human experience, characterized by inevitable failures and imperfections; and (3) Mindfulness, which entails adopting an open, accepting, and nonjudgmental perspective towards one’s own suffering, as opposed to becoming overly identified or consumed by it.

To empirically assess self-compassion, the Self-Compassion Scale (SCS) is the prevailing instrument. It evaluates six distinct dimensions, divided into active elements and potential barriers. The active elements consist of Self-Kindness (SK), Common Humanity (CH), and Mindfulness (MI). These components encapsulate a benevolent attitude towards oneself, the acknowledgment that suffering is an integral part of the human condition, and a mindful awareness of distressing thoughts and emotions. On the other hand, the scale also measures barriers to self-compassion, including Self-Judgment (SJ), Isolation (IS), and Overidentification (OI). These barriers explore the tendencies towards self-criticism, feelings of social isolation, and excessive emotional entanglement in one’s own struggles, respectively [18].

Emerging evidence strongly suggests a negative correlation between stress and self-compassion, as well as a positive association between self-compassion and reduced occupational burnout [19]. In this context, specialized studies have started to explore the impact of self-compassion on the psychological well-being of emergency responders. For instance, Pietrantoni and Prati (2008) [20] found minimal levels of compassion fatigue and burnout but high job satisfaction among a sample of emergency personnel. Extending these findings, Lowery and Cassidy (2022) [21] broadened the scope to include a wider array of first responders such as police officers and firefighters, emphasizing self-compassion as a key factor in promoting well-being. Further, studies that focus specifically on firefighters, like the one by Lv et al. (2023) [22], have identified both self-compassion and maladaptive coping strategies as mediating variables that influence the relationship between stress and occupational burnout.

In light of this evidence, it is plausible to posit that self-compassion could be a key factor in boosting resilience among various professional groups, including emergency responders. However, the existing literature presents a noticeable gap in understanding how individual differences influence this relationship. Specifically, it remains uncertain whether emergency responders who exhibit adaptive resilience are more inclined to adopt self-compassion as a coping mechanism compared to those showing maladaptive resilience patterns. To bridge this gap, the current study seeks to identify the distinct individual differences that shape the interplay between protective and vulnerability factors among emergency responders, with a particular focus on their propensity to use self-compassion as a coping strategy. Our study has two primary objectives: (1) to delineate unique individual difference “profiles” based on distinct configurations of protective and vulnerability factors and examine their associations with different levels of self-compassion; and (2) to determine which specific aspects of self-compassion are most closely linked to maladaptive resilience profiles.

### 1.2. Individual Differences in Resilience among Emergency Responders

To identify distinct resilience profiles among emergency responders who vary in their ability to cope with the vicarious trauma inherent in their profession [23], we evaluated multiple dimensions of individual differences.

#### 1.2.1. Personality Traits

In the context of this study, personality traits perform a dual function: they are not only essential constituents of resilience but also serve as significant predictors for susceptibility to burnout. In this way, they bridge external and internal factors, contributing to a comprehensive understanding of psychological well-being [6,24]. Among these traits, neuroticism emerges as a particularly potent predictor of burnout and plays a crucial role in shaping anxiety-related coping styles like vigilance and cognitive avoidance [25,26]. The impact of neuroticism is double-edged; while higher levels make individuals more susceptible to stress, lower levels can serve as protective factors against PTSD, especially among emergency medical personnel [27].

Conversely, lower levels of extraversion and conscientiousness have been linked with maladaptive coping mechanisms and decreased psychological well-being. Specifically, individuals with lower extraversion often concentrate on the negative aspects of challenging situations and are more prone to engage in emotion-focused coping [28]. Reduced conscientiousness is associated with increased depersonalization and diminished feelings of personal accomplishment [29]. In the specific context of RWs, lower levels of extraversion and higher introversion are correlated with an elevated risk of psychological disorders and PTSD [6,30,31].

High levels of agreeableness, on the other hand, correlate with effective interpersonal relationships and emotional intelligence, and, consequently, lower burnout rates [32]. Instead, the impact of the trait of openness remains inconclusive; existing empirical evidence has not yet conclusively determined its relationship with burnout susceptibility [32,33].

Considering these complex interactions among personality traits, coping strategies, and psychological outcomes, it can be suggested that a multifaceted interplay of high neuroticism and low extraversion, agreeableness, and conscientiousness—though not necessarily openness—may act as a composite “personality marker” for RWs who find it challenging to effectively mobilize internal resources and build resilience against external stressors.

#### 1.2.2. Coping Strategies

Coping refers to the cognitive and behavioral efforts that individuals utilize to manage environmental stressors [34]. Coping strategies are generally classified into two main types: adaptive strategies, such as problem-solving and cognitive reappraisal, and maladaptive strategies, including suppression, rumination, and avoidance. Research indicates that maladaptive coping methods can negatively impact psychological well-being [35,36,37,38]. On the other hand, the lack of adaptive coping mechanisms seems to have less significant repercussions for the development of psychological disorders [37,39].

There is a considerable body of research exploring the links between personality traits and coping styles [28,38]. Specifically, extraversion has been positively correlated with both problem-focused and emotion-focused coping. Neuroticism, conversely, has a negative association with problem-focused and positive strategies like acceptance, but is positively correlated with emotion-focused and avoidance-oriented methods. Agreeableness and openness have shown only a modest association with coping, mainly relating to social support and problem-focused coping. Conscientiousness, however, is strongly tied to problem-focused coping strategies. Moreover, the use of avoidance-oriented coping mechanisms like substance abuse has been negatively correlated with agreeableness and conscientiousness [28,40].

In the specialized setting of emergency responders, ineffective coping strategies have been found to negatively impact resilience. For example, a study focusing on a group of police officers showed that maladaptive coping mechanisms, such as alcohol misuse and rigid behavioral patterns, were linked to an increase in both the chronicity and severity of PTSD symptoms [41].

#### 1.2.3. Perceived Social Support

The presence of both internal and external resources enables individuals to adeptly handle situational challenges, thereby bolstering their resilience against various stressors. In the case of emergency workers, perceived social support stands out as a critical external resource that significantly contributes to reducing burnout. Setti et al. (2016) [42] demonstrated that emergency responders who feel supported by their work environment, particularly by colleagues and supervisors, are likely to experience lower levels of the key components of burnout: emotional exhaustion, depersonalization, and a sense of inefficacy. This evidence is consistent with earlier research linking social support to reduced occurrences of both burnout and post-traumatic symptoms [43].

Various theoretical frameworks further substantiate the importance of social support. These include the Stress-Buffering Hypothesis, which argues that social support can mitigate the effects of stress [44]; the Social Support Deterioration Model, suggesting that dwindling social support can worsen stress-related outcomes [45]; and the Conservation of Resources Model, which posits that maintaining valued resources like social support can safeguard against stress-induced depletion [46].

A meta-analysis conducted by Berger et al. (2012) [47] revealed that emergency responders are significantly more likely to develop PTSD compared to the general population. However, strong social support or social acknowledgment appears to act as a protective factor, making these individuals less susceptible to negative psychological outcomes, including burnout [41,48] and PTSD [49,50].

#### 1.2.4. Life Events

Given the inherently stressful demands of their profession, RWs are frequently exposed to challenging life events, which can indirectly jeopardize their psychological well-being. However, the relationship between such life events and mental health is complex. As underscored by research reviewed by Seery (2011) [51], a U-shaped correlation exists between lifetime exposure to adversity and overall well-being. Specifically, moderate levels of adversity may actually lead to improved mental health outcomes, compared to either extreme adversity or a complete absence of adversity. This complex relationship suggests that the resilience of RWs is shaped not only by the intensity of the stressors they encounter but also by their cumulative life experiences with adversity.

### 1.3. Rationale and Outline of the Study

To achieve the two aims of the current study, we employed Latent Profile Analysis (LPA) to discern distinct clusters of RWs, categorized by their specific protective and risk factors and their resulting outcomes. Subsequently, we probed whether individuals categorized under a ‘maladaptive’ profile exhibited elevated levels of negative dimensions of self-compassion compared to those classified as having an ‘adaptive’ profile.

## 2. Materials and Methods

### 2.1. Participants

A call for participation in the study was advertised in the Italian Red Cross websites for Lombardy and Tuscany. This resulted in a sample of *n* = 791 participating emergency (first responder) rescue-workers (46% female) with 8.77 (8.08) mean years of experience as a rescue worker. Mean age was 39.9 (SD = 13.4); mean length of education was 14.2 years (SD = 2.8); mean rate of activity was 3.7 (SD = 1.01) times per week.

### 2.2. Material

In addition to administering specific questions targeted towards the RW group, we also administered the following scales to both participant groups.

We used the Self-Compassion Scale (SCS) [17] to assess self-compassion across six subscales. In our sample, the Italian version [52] showed good internal consistency, with a total ω = 0.92 and subscale ω values ranging from 0.78 to 0.90;

We used the NEO Five-Factor Inventory (NEO-FFI-60) [53] to assess five personality domains. In our sample, the Italian version [54] showed good internal consistency for most subscales, with ω values of 0.92 for Neuroticism, 0.83 for Extraversion, 0.87 for Conscientiousness, and 0.78 for Openness, but low values for Agreeableness at 0.66;

We used the Coping Orientation to Problems Experienced (COPE) test [55,56] to assess coping strategies, categorized into Active, Emotion-Focused, and Avoidance Coping, with the scoring system proposed by Lyne and Roger (2000) [56]. In our sample, the Italian version [57] showed good reliability, with ω values of 0.89 for Active, 0.77 for Emotion-Focused, and 0.82 for Avoidance Coping;

We used the Multidimensional Scale of Perceived Social Support (MSPSS) [58] to assess perceived social support across family, friends, and significant others, using a seven-point Likert scale. In our sample, the Italian version [59] showed good internal consistency, with ω values of 0.94, 0.96, and 0.95 for the family, friends, and significant others subscales, respectively;

We used the Impact of Event Scale—Revised (IES-R) [60] to measure traumatic stress, covering domains of intrusion, avoidance, and hyperarousal, aligning with DSM-IV PTSD criteria, to assess post-traumatic stress. Its psychometric properties, including good internal consistency and test–retest reliability, are well-established [61]. In our sample, IES-R showed high reliability: total ω = 0.94, and, for sub-scales, ω = 0.91 for intrusion, ω = 0.82 for avoidance, and ω = 0.87 for hyperarousal.

### 2.3. Procedure

#### 2.3.1. Quality Check

To ensure participant engagement [62], we utilized a triad of metrics: the Longstring Index to identify automated or disengaged responses; Within-Person Variance to gauge answer uniformity as a measure of attentiveness; and Bogus Items to flag careless agreement. Using these criteria, 7% of participants were deemed inattentive and excluded from the final analyses.

#### 2.3.2. Data Analysis

In our study, Latent Profile Analysis (LPA) was employed to classify RWs into distinct subgroups based on metrics such as personality traits, coping styles, and stress indicators (see also [63]). This technique aims to optimize within-group homogeneity and between-group heterogeneity. Analyses were conducted in MPLUS 8.6 and R, with Bayesian regression models further interpreting LPA outcomes via R’s brms package. Regularizing priors enhanced model stability, and 95% credible intervals were used for parameter estimation [64].

#### 2.3.3. Power Analysis

In their investigation, Tekle et al. (2016) [65] utilized Monte Carlo bootstrap simulations to ascertain the relationship between sample size and statistical power, particularly in the context of a two-class model with low class separation. Their findings indicate that a sample size of 600, combined with 10 indicators, is sufficient to achieve a statistical power of 1.00. This evidence substantiates the suitability of the sample size employed in the present study, affirming its adequacy for the intended statistical analysis.

## 3. Results

Table 1 presents the descriptive statistics for the variables under investigation. We used LPA with 1 to 10 profiles and 1000 initial value sets to improve robustness. Following Akogul and Erisoglu (2017) [66], an Analytic Hierarchy Process and multiple fit indices were used for model selection. Model 6 emerged as optimal, allowing variability in variances and covariances and identifying a two-class structure.

We assessed class precision using entropy and the Bootstrap Likelihood Ratio Test (BLRT), with entropy values over 0.6 indicating adequate class separation [67]. Our model showed high class membership probabilities (0.89 and 0.94) and balanced case proportions (0.44 and 0.56), confirming the reliability of our class assignments. Table A1 in Appendix A presents the fit indices for the evaluated model configurations.

The solution identifying two profiles differentiates between a high-resilience group (*n* = 408) and a low-resilience group (*n* = 343), as shown in Table 2. These profiles were labeled as “high resilience” and “low resilience” to encapsulate their distinct characteristics with respect to stress, burnout, and PTSD. In particular, the “high resilience” profile is characterized by elevated levels of metrics commonly viewed as protective factors against stress-related outcomes, in addition to lower values for variables identified as risk factors.

When contrasting Profile 1 (high resilience) with Profile 2 (low resilience), the following reliable differences were observed across several psychological and behavioral measures: Neuroticism exhibited reliably lower levels in Profile 1, with a coefficient (β) of −7.11 and a 95% Credible Interval (CI) ranging from −8.34 to −5.90; Extraversion showed reliably higher levels in Profile 1, with a β value of 4.04 and a 95% CI of [3.15, 4.94]; Agreeableness was reliably higher in Profile 1, marked by a β of 3.04 and a 95% CI of [2.25, 3.84]; Conscientiousness also registered reliably higher levels in Profile 1, evidenced by a β value of 2.69 and a 95% CI of [1.75, 3.61]; Avoidance Coping displayed reliably lower levels in Profile 1, characterized by a β of −2.46 and a 95% CI of [−3.34, −1.63]; Perceived social support was reliably higher in Profile 1, as indicated by a β of 3.12 and a 95% CI of [1.60, 5.24]; IES-R levels were reliably lower in Profile 1, demonstrated by a β of −4.15 and a 95% CI of [−6.27, −2.54].

**Figure 1:**
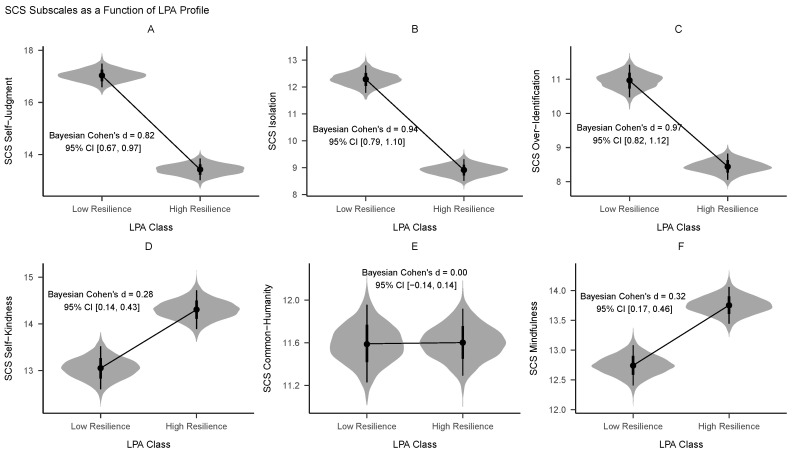
Violin plot comparisons of posterior distributions for the two LPA classes across each subscale within the Self-Compassion Scale. (**A**) Self-Judgment subscale. (**B**) Isolation subscale. (**C**) Over-Identification subscale. (**D**) Self-Kindness subscale. (**E**) Common-Humanity subscale. (**F**) Mindfulness subscale.

The high-resilience group exhibited an elevated total score on the Self-Compassion Scale (β= 12.71; 95% CI = [10.50, 14.95], Cohen’s *d* = 0.87, 95% CI = [0.72, 1.03]). As shown in Figure 1, individuals within the low-resilience profile consistently registered higher scores across all three negative facets of Self-Compassion—namely, Self-Judgment, Isolation, and Overidentification. In each instance, the magnitude of these differences was large, as indicated by Cohen’s *d*. Conversely, the low-resilience group displayed comparatively reduced levels of Self-Kindness and Mindfulness relative to the high-resilience group; however, these differences were of a smaller magnitude, based on Cohen’s *d*.

Engagement metrics, such as rate of activity and time elapsed since last training, showed no reliable differences between the low-resilience (3.60/month, 8.82 months) and high-resilience profiles (3.62/month, 8.28 months; $\beta_{\mathrm{diff}}$ = 0.04, −0.06; 95% CI = [−0.22, 0.31], [−0.22, 0.09]).

In predicting membership to the maladaptive profile, Bayesian logistic regression estimated the probabilities as 0.378 for drivers, 0.463 for team leaders, and 0.488 for team members. When contrasted with drivers, the estimates for team leaders and team members (on a logit scale) were $\beta_{\mathrm{diff}}$ = −0.351, (95% CI = [−0.739, 0.051]) and $\beta_{\mathrm{diff}}$ = −0.450 (95% CI = [−0.823, −0.043]), respectively.

In a Bayesian regression model assessing IES-R scores as a function of job qualification, the estimated scores ($\hat{Y}$) were 17.9 for drivers, 19.1 for team leaders, and 19.4 for team members. Relative to drivers, the $\beta_{\mathrm{diff}}$ estimates for team leaders and team members were −1.165 (95% CI = [−2.45, −0.043]) and −1.448 (95% CI = [−2.73, −0.193]), respectively.

## 4. Discussion

Our study explored individual differences in protective and risk factors among RWs, linking these to self-compassion levels. Through a person-centered approach, we identified two distinct psychological profiles within the RW population: one that is adaptively resilient and another that is maladaptively vulnerable. These profiles were uniquely defined by a combination of personality traits, coping strategies, significant life events, and social support networks.

The resilient profile is marked by lower levels of Neuroticism and higher levels of Extraversion, Agreeableness, and Conscientiousness. It also shows minimal reliance on Avoidance Coping strategies, fewer life events as measured by the IES-R scale, and a strong perception of social support. On the contrary, the vulnerable profile exhibits the opposite characteristics, indicating a heightened risk for poor mental health outcomes.

The psychological attributes that distinguish the two profiles in our study are well-supported by the existing literature. Specifically, Neuroticism and Extraversion have been prominently highlighted as substantial contributors to resilience [68,69,70,71,72]. Our study further substantiates the roles that Agreeableness and Conscientiousness play in resilience, echoing prior findings [73]. While some studies have suggested a role for Openness in resilience (see [73]), this trait did not act as a differentiating characteristic within our sample.

Additionally, our findings are in line with prior studies that highlight the adverse long-term effects of resorting to avoidant coping mechanisms, particularly in the face of traumatic stress [74,75]. Notably, we found that RWs characterized by resilient profiles exhibited a lower propensity to engage in such detrimental coping strategies.

Resilience has been recognized as a mediating factor between exposure to traumatic stress and the subsequent development of PTSD symptoms. Specifically, in a study by Lee et al. (2014) [76], firefighters exposed to similar degrees of traumatic stress demonstrated varying outcomes based on their resilience levels. Those with higher resilience were more effectively shielded from both the immediate and long-term consequences of such stress compared to their less resilient peers. Consistent with these findings, our results indicates that individuals falling under the “vulnerable” psychological profile showed elevated scores on the Impact of Event Scale—Revised (IES-R), indicating a heightened vulnerability to traumatic stress. Moreover, our results further underline the significance of perceived social support as a crucial element in resilience and mental well-being, reinforcing earlier research that touted its benefits in stress adaptation [77].

The findings from our latent profile analysis corroborated the relationship between risk and protective factors among RWs, aligning with similar trends observed in other populations. The novel contribution of our study resides in the use of self-compassion as an external criterion to differentiate between profiles. Employing this approach, we identified a marked difference between the two psychological profiles. Specifically, RWs categorized within the ‘Maladaptively Vulnerable’ profile displayed heightened inclinations toward self-judgment, social isolation, and over-identification.

Self-judgment and its close affiliate, self-blame, constitute significant barriers to self-compassion. According to the existing literature, self-blame serves as a prevalent coping mechanism among individuals with diminished resilience [78]. This cognitive–emotional schema, wherein individuals attribute adversities to their own shortcomings, aligns closely with the construct of self-judgment. Such maladaptive coping mechanisms starkly contrast with adaptive, task-oriented strategies like problem-solving and positive cognitive reappraisal, which are often employed by individuals with higher resilience levels.

Social isolation serves as a notable barrier to self-compassion and is fundamentally interconnected with the inherent human need for social bonds. The lack of such affiliations not only exacerbates the likelihood of adverse health repercussions but also has a disproportionate impact on individuals exhibiting vulnerable psychological profiles [79]. Our research corroborates these findings.

Finally, overidentification with personal suffering presents a dual threat to psychological well-being, as it is positively correlated with compassion fatigue and negatively with resilience. This excessive focus on personal distress, often manifested as rumination, has been shown to be detrimental in various contexts, including among healthcare professionals during the COVID-19 pandemic [80]. Our findings corroborates these observations, underscoring the adverse impacts of overidentification on mental health.

A critical question that emerges is why the resilient and vulnerable profiles among RWs show pronounced differences primarily in barriers to self-compassion—namely, self-judgment, social isolation, and overidentification—rather than in the proactive and positive facets of self-compassion. This question ties into an ongoing scholarly debate concerning the relationship between self-compassion and psychopathology. While a considerable body of research suggests that self-compassion, as quantified by the aggregate score on the Self-Compassion Scale (SCS), serves as a protective factor against mental health issues [19], there has been some debate regarding the scale’s inclusion of what are termed “uncompassionate” dimensions: self-judgment, social isolation, and overidentification. Critics argue that these components are more indicative of vulnerability and psychopathological symptoms rather than aspects of self-compassion (e.g., [81]). This debate becomes more complicated when the SCS’s total score, which incorporates both the presence of positive and the absence of negative traits, is used to predict mental health outcomes. Employing the scale in this manner introduces a “measurement confounding” issue, which in turn leads to circular reasoning.

Our data contribute to this debate by indicating that, while both resilient and vulnerable profiles do differ in terms of positive self-compassion aspects (namely, self-kindness and mindfulness), these differences are relatively minor. On the other hand, the gaps in the uncompassionate dimensions are substantially larger. This finding lends empirical support to the critiques posited by Muris and Otgaar (2020) [82] and others [83,84], suggesting that these “uncompassionate” dimensions may indeed be more relevant indicators of mental health vulnerabilities.

Concerning the issue of psychological support, our findings indicate that targeted interventions could be especially beneficial for RWs at a higher risk of experiencing emotional and psychological stress. In terms of intervention strategies, two divergent approaches have been put forth: one advocates for holistically enhancing self-compassion, while the other focuses on mitigating its negative aspects. This divergence in treatment modalities remains a subject of active academic discussion [19].

When considering the problem of identifying those in need of specialized support, our result indicate that conventional external metrics, such as rate of activity and level of engagement, are inadequate for reliably differentiating between resilient and vulnerable profiles. This suggests that relying solely on such observable factors may be insufficient for detecting at-risk individuals, despite plausible assumptions that lower engagement might correlate with poorer mental health outcomes (e.g., [85]). In contrast, our study suggests that a more targeted approach, one that focuses on individual variations in psychological dimensions, would offer a more reliable method for identifying those at heightened risk for mental health issues.

Our study also uncovers a link between job roles and resilience profiles among RWs. We found that high-resilience profiles are more common among drivers, followed by team leaders, and, lastly, team members. This hierarchy also correlates with self-compassion scores, with drivers scoring the highest. This tiered pattern can be understood in the context of the different job responsibilities associated with each role. Drivers, who generally have less direct exposure to human suffering, face fewer emotional and ethical challenges. This is reflected in their higher prevalence in the adaptively resilient category and their relatively elevated self-compassion scores. On the other hand, team members, who often confront human suffering more directly, are more prone to exhibit maladaptive resilience profiles and lower self-compassion scores. This is consistent with existing literature indicating that roles requiring higher emotional labor are associated with decreased psychological well-being [86].

Adding weight to this observation is the influence of life events in shaping these maladaptive profiles. Drivers, who are generally less exposed to the direct emotional toll of their work, are less likely to fall into maladaptive categories. This suggests that the severity of indirect trauma exposure, coupled with individual psychological vulnerabilities, can be a significant precursor to maladaptive psychological responses. In practical terms, our study reveals that this often translates into greater reliance on unhealthy emotional regulation strategies, including maladaptive coping mechanisms and increased tendencies for self-judgment, over-identification, and social isolation.

While our study offers valuable insights into the psychological profiles of RWs and suggests actionable intervention strategies, it is important to acknowledge its limitations. Firstly, the sample size may not be representative of the larger population of RWs, which could limit the generalizability of our findings. Additionally, our study population might lack diversity in terms of geography, gender, or ethnic background, further restricting the applicability of the results. Our study employs a cross-sectional design, which captures a snapshot of psychological profiles but cannot provide insights into their evolution over time. Longitudinal studies would be needed to understand the temporal dynamics of resilience and self-compassion among RWs. The use of self-reported measures for variables like self-compassion and coping mechanisms may introduce biases, such as social desirability or recall bias. Objective measures or third-party evaluations could provide a more balanced view. The study focuses mainly on self-compassion as the criterion variable for differentiating between adaptive and maladaptive profiles. The inclusion of other psychological variables could offer a more comprehensive understanding of resilience among RWs. Although our study indicates that external metrics like rate of activity and degree of engagement are insufficient for identifying at-risk individuals, we did not explore other potential external indicators that could be more predictive. Our findings touch upon ongoing debates in the field, such as the most effective way to enhance self-compassion. Additional research is needed to ascertain the relative efficacy of different intervention strategies. Further, understanding the degree to which enhancing self-compassion can act as a catalyst for overall resilience in RWs is a critical yet unresolved issue.

The present study also had notable strengths relative to other studies that have examined the role of self-compassion in first responders. For example, this is the first study that uses a person-based approach alongside a broad battery of psychological outcomes. This methodology allowed us to evaluate the independent contributions of each of these protective and risk factors and to demonstrate unique patterns of associations with different profiles of individual differences.

## 5. Conclusions

In conclusion, notwithstanding its limitations, our study suggests the potential value of rethinking pre-employment strategies to better identify candidates who may be well-suited to the emotional and ethical challenges of rescue work. Additionally, our findings point to the possible advantages of developing targeted interventions designed to bolster self-compassion among those RWs who appear to be most vulnerable.

## Figures and Tables

**Table 1:** Descriptive statistics (*n* = 746).

Variable	Mean	Std. Err.
Agreeableness	32.48	0.21
Conscientiousness	35.77	0.24
Extraversion	33.16	0.25
Neuroticism	19.10	0.32
Openness	30.09	0.23
Active Coping	79.44	0.38
Avoidance Coping	34.91	0.26
Emotion-Focused Coping	29.99	0.26
IES-R	18.46	0.57
MSPSS	55.76	0.47

*Note:* Agreeableness, Conscientiousness, Extraversion, Neuroticism, Openness: Subscales of the NEO Five-Factor Inventory; Active Coping, Avoidance Coping, Emotion-Focused Coping: Subscales of the Coping Orientation to Problems Experienced; IES-R = The Impact of Event Scale—Revised; MSPSS = Multidimensional Scale of Perceived Social Support.

**Table 2:** Latent profile average means and standard error.

Parameter	Estimate (Class 1)	SE (Class 1)	Estimate (Class 2)	SE (Class 2)
Agreeableness	0.216	0.066	−0.243	0.079
Conscientiousness	0.181	0.058	−0.203	0.069
Extraversion	0.301	0.060	−0.338	0.070
Neuroticism	−0.369	0.063	0.414	0.080
Openness	0.017	0.063	−0.019	0.073
Active Coping	−0.011	0.064	0.013	0.071
Avoidance Coping	−0.299	0.048	0.336	0.081
Emotion-Focused Coping	−0.069	0.064	0.077	0.075
IES-R	−0.503	0.040	0.564	0.081
MSPSS	0.403	0.077	−0.452	0.082

## Data Availability

The data and R code used in our analyses are available at the Open Science Framework: https://osf.io/fp3yn/?view_only=5ad872209d8843e6ae3def4f8c27978f (accessed on 21 December 2023). This study was not pre-registered.

## Appendix A

**Table A1:** Model fit for 1 through 10 profile solutions tested for the RW sample.

Model	Classes	AIC	BIC	Entropy	$p_{\min}$	$p_{\max}$	$n_{\min}$	$n_{\max}$	BLRT_p
1	1	21,342.45	21,434.88	1.00	1.00	1.00	1.00	1.00	
1	2	20,740.67	20,883.93	0.71	0.88	0.94	0.36	0.64	0.01
1	3	20,460.08	20,654.18	0.75	0.81	0.93	0.19	0.56	0.01
1	4	20,335.36	20,580.29	0.73	0.84	0.86	0.11	0.36	0.01
1	5	20,199.94	20,495.71	0.73	0.79	0.87	0.08	0.32	0.01
1	6	20,148.07	20,494.68	0.73	0.73	0.88	0.07	0.36	0.01
1	7	20,080.74	20,478.18	0.76	0.72	0.88	0.05	0.37	0.01
1	8	20,014.89	20,463.17	0.77	0.71	0.90	0.03	0.36	0.01
1	9	19,965.51	20,464.63	0.76	0.76	0.88	0.04	0.27	0.01
1	10	19,948.33	20,498.28	0.75	0.69	0.92	0.02	0.22	0.01
6	1	19,883.12	20,183.51	1.00	1.00	1.00	1.00	1.00	
6	2	19,509.40	20,114.80	0.70	0.89	0.94	0.44	0.56	0.01
6	3	19,416.00	20,326.42	0.73	0.88	0.89	0.23	0.39	0.01
6	4	19,366.48	20,581.91	0.80	0.88	0.90	0.09	0.40	0.01
6	5	19,360.48	20,880.92	0.79	0.83	0.92	0.11	0.37	0.09
6	6	19,313.54	21,138.99	0.82	0.84	0.98	0.07	0.29	0.01
6	7	19,374.46	21,504.93	0.82	0.83	0.98	0.07	0.28	0.96
6	8	19,285.55	21,721.03	0.85	0.83	0.99	0.07	0.22	0.01
6	9	19,275.58	22,016.07	0.87	0.88	0.97	0.07	0.18	0.15
6	10	19,342.60	22,388.10	0.88	0.87	0.98	0.06	0.14	1.00

## References

[1] Chatzea V.-E., Sifaki-Pistolla D., Vlachaki S.-A., Melidoniotis E., Pistolla G. (2018). PTSD, burnout and well-being among rescue workers: Seeking to understand the impact of the european refugee crisis on rescuers. Psychiatry Res..

[2] Cieslak R., Shoji K., Douglas A., Melville E., Luszczynska A., Benight C.C. (2014). A meta-analysis of the relationship between job burnout and secondary traumatic stress among workers with indirect exposure to trauma. Psychol. Serv..

[3] Joinson C. (1992). Coping with compassion fatigue. Nursing.

[4] Geronazzo-Alman L., Eisenberg R., Shen S., Duarte C.S., Musa G.J., Wicks J., Fan B., Doan T., Guffanti G., Bresnahan M. (2017). Cumulative exposure to work-related traumatic events and current post-traumatic stress disorder in new york city’s first responders. Compr. Psychiatry.

[5] Tahernejad S., Ghaffari S., Ariza-Montes A., Wesemann U., Farahmandnia H., Sahebi A. (2023). Post-traumatic stress disorder in medical workers involved in earthquake response: A systematic review and meta-analysis. Heliyon.

[6] Mao X., Hu X., Loke A.Y. (2022). A concept analysis on disaster resilience in rescue workers: The psychological perspective. Disaster Med. Public Health Prep..

[7] McDonald M.A., Yang Y., Lancaster C.L. (2022). The association of distress tolerance and mindful awareness with mental health in first responders. Psychol. Serv..

[8] Paton D., Smith L., Violanti J. (2000). Disaster response: Risk, vulnerability and resilience. Disaster Prev. Manag. Int. J..

[9] Beck A.T. (2016). The Self in Understanding and Treating Psychological Disorders.

[10] MacBeth A., Gumley A. (2012). Exploring compassion: A meta-analysis of the association between self-compassion and psychopathology. Clin. Psychol. Rev..

[11] Wilson A.C., Mackintosh K., Power K., Chan S.W. (2019). Effectiveness of self-compassion related therapies: A systematic review and meta-analysis. Mindfulness.

[12] Wong C.C.Y., Yeung N.C. (2017). Self-compassion and posttraumatic growth: Cognitive processes as mediators. Mindfulness.

[13] Alex D.A., Klein S. (2009). First responders after disasters: A review of stress reactions, at-risk, vulnerability, and resilience factors. Prehospital Disaster Med..

[14] Scuri S., Petrelli F., Nguyen T., Grappasonni I. (2019). Training to improve resilience and coping to monitor PTSD in rescue workers. J. Prev. Med. Hyg..

[15] Ludick M., Figley C.R. (2017). Toward a mechanism for secondary trauma induction and reduction: Reimagining a theory of secondary traumatic stress. Traumatology.

[16] Mao X., Fung O.W., Hu X., Loke A.Y. (2022). Characteristics of resilience among disaster rescue workers: A systematic review. Disaster Med. Public Health Prep..

[17] Neff K.D. (2003). Self-compassion: An alternative conceptualization of a healthy attitude toward oneself. Self Identity.

[18] Neff K.D. (2022). The differential effects fallacy in the study of self-compassion: Misunderstanding the nature of bipolar continuums. Mindfulness.

[19] Neff K.D. (2023). Self-compassion: Theory, method, research, and intervention. Annu. Rev. Psychol..

[20] Pietrantoni L., Prati G. (2008). Resilience among first responders. Afr. Health Sci..

[21] Lowery A., Cassidy T. (2022). Health and well-being of first responders: The role of psychological capital, self-compassion, social support, relationship satisfaction, and physical activity. J. Workplace Behav. Health.

[22] Lv G., Li J., Xu Q., Zhang H., Wu W., Fan X., Wang Z., Liu H. (2023). The influence of firefighters’ perceived stress on job burnout: A moderated mediation model. Curr. Psychol..

[23] Palm K.M., Polusny M.A., Follette V.M. (2004). Vicarious traumatization: Potential hazards and interventions for disaster and trauma workers. Prehospital Disaster Med..

[24] Swider B.W., Zimmerman R.D. (2010). Born to burnout: A meta-analytic path model of personality, job burnout, and work outcomes. J. Vocat. Behav..

[25] Bianchi R. (2018). Burnout is more strongly linked to neuroticism than to work-contextualized factors. Psychiatry Res..

[26] Jung S., Sindermann C., Yang H., Elhai J.D., Montag C. (2022). Anxiety-related coping styles and individual differences in primary emotional systems against the background of affective neuroscience theory: A study using samples from germany and china. Trends Psychol..

[27] Mirhaghi A., Mirhaghi M., Oshio A., Sarabian S. (2016). Systematic review of the personality profile of paramedics: Bringing evidence into emergency medical personnel recruitment policy. Eurasian J. Emerg. Med..

[28] Connor-Smith J.K., Flachsbart C. (2007). Relations between personality and coping: A meta-analysis. J. Personal. Soc. Psychol..

[29] Kokkinos C.M. (2007). Job stressors, personality and burnout in primary school teachers. Br. J. Educ. Psychol..

[30] Liao S.-C., Lee M.-B., Lee Y.-J., Weng T., Shih F.-Y., Ma M.H. (2002). Association of psychological distress with psychological factors in rescue workers within two months after a major earthquake. J. Formos. Med. Assoc..

[31] Naz M., Saleem S., Mahmood Z. (2010). Development of indigenous resilience scale for rescue 1122 workers. Pak. J. Psychol. Res..

[32] Angelini G. (2023). Big five model personality traits and job burnout: A systematic literature review. BMC Psychol..

[33] Răducu C.-M., Stănculescu E. (2022). Personality and socio-demographic variables in teacher burnout during the COVID-19 pandemic: A latent profile analysis. Sci. Rep..

[34] Lazarus S.R., Folkman S. (1984). Stress, Appraisal, and Coping.

[35] Joormann J., Stanton C.H. (2016). Examining emotion regulation in depression: A review and future directions. Behav. Res. Ther..

[36] Liu D.Y., Thompson R.J. (2017). Selection and implementation of emotion regulation strategies in major depressive disorder: An integrative review. Clin. Psychol. Rev..

[37] Moritz S., Jahns A.K., Schröder J., Berger T., Lincoln T.M., Klein J.P., Göritz A.S. (2016). More adaptive versus less maladaptive coping: What is more predictive of symptom severity? Development of a new scale to investigate coping profiles across different psychopathological syndromes. J. Affect. Disord..

[38] Sica C., Latzman R.D., Caudek C., Cerea S., Colpizzi I., Caruso M., Giulini P., Bottesi G. (2021). Facing distress in coronavirus era: The role of maladaptive personality traits and coping strategies. Personal. Individ. Differ..

[39] Aldao A., Nolen-Hoeksema S. (2012). When are adaptive strategies most predictive of psychopathology?. J. Abnorm. Psychol..

[40] Afshar-Oromieh A., Avtzi E., Giesel F.L., Holl-Letz T., Linhart H.G., Eder M., Eisenhut M., Boxler S., Hadaschik B.A., Kratochwil C. (2015). The diagnostic value of PET/CT imaging with the 68 ga-labelled PSMA ligand HBED-CC in the diagnosis of recurrent prostate cancer. Eur. J. Nucl. Med. Mol. Imaging.

[41] Marmar C.R., McCaslin S.E., Metzler T.J., Best S., Weiss D.S., Fagan J., Liberman A., Pole N., Otte C., Yehuda R. (2006). Predictors of posttraumatic stress in police and other first responders. Ann. N. Y. Acad. Sci..

[42] Setti I., Lourel M., Argentero P. (2016). The role of affective commitment and perceived social support in protecting emergency workers against burnout and vicarious traumatization. Traumatology.

[43] Armstrong-Stassen M. (2004). The influence of prior commitment on the reactions of layoff survivors to organizational downsizing. J. Occup. Health Psychol..

[44] Cohen S., Wills T.A. (1985). Stress, social support, and the buffering hypothesis. Psychol. Bull..

[45] Norris F.H., Kaniasty K. (1996). Received and perceived social support in times of stress: A test of the social support deterioration deterrence model. J. Personal. Soc. Psychol..

[46] Hobfoll S.E. (1989). Conservation of resources: A new attempt at conceptualizing stress. Am. Psychol..

[47] Berger W., Coutinho E.S.F., Figueira I., Marques-Portella C., Luz M.P., Neylan T.C., Marmar C.R., Mendlowicz M.V. (2012). Rescuers at risk: A systematic review and meta-regression analysis of the worldwide current prevalence and correlates of PTSD in rescue workers. Soc. Psychiatry Psychiatr. Epidemiol..

[48] Thormar S.B., Sijbrandij M., Gersons B.P., Van de Schoot R., Juen B., Karlsson T., Olff M. (2016). PTSD symptom trajectories in disaster volunteers: The role of self-efficacy, social acknowledgement, and tasks carried out. J. Trauma. Stress.

[49] Eriksson C.B., Lopes Cardozo B., Foy D.W., Sabin M., Ager A., Snider L., Scholte W.F., Kaiser R., Olff M., Rijnen B. (2013). Predeployment mental health and trauma exposure of expatriate humanitarian aid workers: Risk and resilience factors. Traumatology.

[50] Pietrzak R.H., Feder A., Singh R., Schechter C.B., Bromet E.J., Katz C., Reissman D., Ozbay F., Sharma V., Crane M. (2014). Trajectories of PTSD risk and resilience in world trade center responders: An 8-year prospective cohort study. Psychol. Med..

[51] Seery M.D. (2011). Resilience: A silver lining to experiencing adverse life events?. Curr. Dir. Psychol. Sci..

[52] Veneziani C.A., Fuochi G., Voci A. (2017). Self-compassion as a healthy attitude toward the self: Factorial and construct validity in an italian sample. Personal. Individ. Differ..

[53] Costa P.T., McCrae R.R. (1992). Normal personality assessment in clinical practice: The NEO personality inventory. Psychol. Assess..

[54] Caprara G.V., Barbaranelli C., Guido G. (2001). Brand personality: How to make the metaphor fit?. J. Econ. Psychol..

[55] Carver C.S., Scheier M.F., Weintraub J.K. (1989). Assessing coping strategies: A theoretically based approach. J. Personal. Soc. Psychol..

[56] Lyne K., Roger D. (2000). A psychometric re-assessment of the COPE questionnaire. Personal. Individ. Differ..

[57] Sica C., Novara C., Dorz S., Sanavio E. (1997). Coping strategies: Evidence for cross-cultural differences? A preliminary study with the italian version of coping orientations to problems experienced (COPE). Personal. Individ. Differ..

[58] Zimet G.D., Dahlem N.W., Zimet S.G., Farley G.K. (1988). The multidimensional scale of perceived social support. J. Personal. Assess..

[59] Prezza M., Principato M.C., Prezza M., Santinello M. (2002). La rete sociale e il sostegno sociale. Conoscere la Comunità.

[60] Weiss D.S. (2007). The impact of event scale: Revised. Cross-Cultural Assessment of Psychological Trauma and PTSD.

[61] Craparo G., Faraci P., Rotondo G., Gori A. (2013). The impact of event scale–revised: Psychometric properties of the italian version in a sample of flood victims. Neuropsychiatr. Dis. Treat..

[62] Ward M., Meade A.W. (2023). Dealing with careless responding in survey data: Prevention, identification, and recommended best practices. Annu. Rev. Psychol..

[63] Ullrich-French S., Cox A.E. (2020). The use of latent profiles to explore the multi-dimensionality of self-compassion. Mindfulness.

[64] Kruschke J.K., Liddell T.M. (2018). Bayesian data analysis for newcomers. Psychon. Bull. Rev..

[65] Tekle F.B., Gudicha D.W., Vermunt J.K. (2016). Power analysis for the bootstrap likelihood ratio test for the number of classes in latent class models. Adv. Data Anal. Classif..

[66] Akogul S., Erisoglu M. (2017). An approach for determining the number of clusters in a model-based cluster analysis. Entropy.

[67] Asparouhov T., Muthén B. (2014). Auxiliary variables in mixture modeling: Three-step approaches using m plus. Struct. Equ. Model. Multidiscip. J..

[68] Friborg O., Barlaug D., Martinussen M., Rosenvinge J.H., Hjemdal O. (2005). Resilience in relation to personality and intelligence. Int. J. Methods Psychiatr. Res..

[69] Campbell-Sills L., Cohan S.L., Stein M.B. (2006). Relationship of resilience to personality, coping, and psychiatric symptoms in young adults. Behav. Res. Ther..

[70] Jeronimus B.F., Ormel J., Aleman A., Penninx B.W., Riese H. (2013). Negative and positive life events are associated with small but lasting change in neuroticism. Psychol. Med..

[71] Ogle C.M., Rubin D.C., Siegler I.C. (2014). Changes in neuroticism following trauma exposure. J. Personal..

[72] Sarubin N., Wolf M., Giegling I., Hilbert S., Naumann F., Gutt D., Jobst A., Sabaß L., Falkai P., Rujescu D. (2015). Neuroticism and extraversion as mediators between positive/negative life events and resilience. Personal. Individ. Differ..

[73] Oshio A., Taku K., Hirano M., Saeed G. (2018). Resilience and big five personality traits: A meta-analysis. Personal. Individ. Differ..

[74] Hetzel-Riggin M.D., Meads C.L. (2016). Interrelationships among three avoidant coping styles and their relationship to trauma, peritraumatic distress, and posttraumatic stress disorder. J. Nerv. Ment. Dis..

[75] Korem N., Ben-Zion Z., Spiller T.R., Duek O.A., Harpaz-Rotem I., Pietrzak R.H. (2023). Correlates of avoidance coping in trauma-exposed US military veterans: Results from the national health and resilience in veterans study. J. Affect. Disord..

[76] Lee J.-S., Ahn Y.-S., Jeong K.-S., Chae J.-H., Choi K.-S. (2014). Resilience buffers the impact of traumatic events on the development of PTSD symptoms in firefighters. J. Affect. Disord..

[77] Pejičić M., Ristić M., Anelković V. (2018). The mediating effect of cognitive emotion regulation strategies in the relationship between perceived social support and resilience in postwar youth. J. Community Psychol..

[78] Ching S.S.Y., Cheung K., Hegney D., Rees C.S. (2020). Stressors and coping of nursing students in clinical placement: A qualitative study contextualizing their resilience and burnout. Nurse Educ. Pract..

[79] Holt-Lunstad J., Steptoe A. (2022). Social isolation: An underappreciated determinant of physical health. Curr. Opin. Psychol..

[80] Ruiz-Fernández M.D., Ramos-Pichardo J.D., Ibáñez-Masero O., Carmona-Rega M.I., Sánchez-Ruiz M.J., Ortega-Galán Á.M. (2021). Professional quality of life, self-compassion, resilience, and empathy in healthcare professionals during COVID-19 crisis in spain. Res. Nurs. Health.

[81] Muris P., van den Broek M., Otgaar H., Oudenhoven I., Lennartz J. (2018). Good and bad sides of self-compassion: A face validity check of the self-compassion scale and an investigation of its relations to coping and emotional symptoms in non-clinical adolescents. J. Child Fam. Stud..

[82] Muris P., Petrocchi N. (2017). Protection or vulnerability? A meta-analysis of the relations between the positive and negative components of self-compassion and psychopathology. Clin. Psychol. Psychother..

[83] Muris P., Otgaar H. (2020). The process of science: A critical evaluation of more than 15 years of research on self-compassion with the self-compassion scale. Mindfulness.

[84] Pfattheicher S., Geiger M., Hartung J., Weiss S., Schindler S. (2017). Old wine in new bottles? The case of self–compassion and neuroticism. Eur. J. Personal..

[85] Mauno S., Ruokolainen M., Kinnunen U., De Bloom J. (2016). Emotional labour and work engagement among nurses: Examining perceived compassion, leadership and work ethic as stress buffers. J. Adv. Nurs..

[86] Zapf D. (2002). Emotion work and psychological well-being: A review of the literature and some conceptual considerations. Hum. Resour. Manag. Rev..

